# Impacts of blood culture-negative status on outcomes of infectious endocarditis patients with nonsurgical and surgical intervention

**DOI:** 10.1097/MD.0000000000048760

**Published:** 2026-05-08

**Authors:** Jing-bin Huang, Zhai Huang, Chang-chao Lu, Zhao-ke Wen

**Affiliations:** a Department of Cardiothoracic Surgery, The People’s Hospital of Guangxi Zhuang Autonomous Region, and Guangxi Academy of Medical Sciences, Nanning, Guangxi, China; b Intensive Care Unit, The People’s Hospital of Guangxi Zhuang Autonomous Region, and Guangxi Academy of Medical Sciences, Nanning, Guangxi, China.

**Keywords:** blood culture-negative, infectious endocarditis, nonsurgical, surgical intervention

## Abstract

This study aims to clarify impacts of blood culture-negative on results of infectious endocarditis (IE) patients with nonsurgical and surgical intervention. This retrospective study was done to investigate patients with IE during study period at our hospital. There were 720 blood culture-negative endocarditis patients (35.7%). In-hospital mortality, aortic regurgitation ≥ 4 cm^2^, mitral regurgitation (MR) ≥ 4 cm^2^, vegetation size ≥ 10 mm, time between symptoms and admission ≥ 2 months, mechanical ventilation length, rate of surgical patients, and acute kidney injury postoperative in blood culture-negative group were significantly higher than those in blood culture-positive group. Time between symptoms and admission ≥ 2 months, vegetation size ≥ 10 mm, aortic regurgitation ≥ 4 cm^2^, MR ≥ 4 cm^2^ vegetations on aortic valve, and vegetations of congenital heart disease were found to be related to blood culture-negative. Univariate and multivariate analysis indicated that blood culture-negative is statistically significantly related to in-hospital mortality, aortic regurgitation ≥ 4 cm^2^, MR ≥ 4 cm^2^, vegetation size ≥ 10 mm, and mechanical ventilation length ≥ 72 hours, respectively. In our investigation, blood culture-negative status is associated with higher in-hospital mortality and more clinical complications including aortic regurgitation ≥ 4 cm^2^, MR ≥ 4 cm^2^, vegetation size ≥ 10 mm, and mechanical ventilation length ≥ 72 hours in IE.

## 1. Introduction

Infectious endocarditis (IE) is a kind of infection of cardiac valve and inner layer of cardiac cavity. It is still a dangerous disease. The mortality rate within 1 year is close to 30%, and the global incidence rate of IE is still rising. The mortality rate of IE is high globally, and its diagnosis is still a grave challenge. Up to 30% or even more cases of IE have been confirmed as blood culture-negative, indicating that the pathogen has not yet been identified. Blood culture-negative endocarditis remains a grave challenge in selecting the best antimicrobial regimen for this fatal infection and should be deal with targeted and effective regimens as much as possible.^[1-6]^ Timely diagnosis, identification of pathogenic microorganisms, and targeted antimicrobial treatment can evidently affect the prognosis of diseases, thereby affecting the health status of patients.^[7-9]^

It has been demonstrated that blood culture-negative endocarditis is related to delayed diagnosis, worsening prognosis and course, as well as more intraoperative and postoperative complications. The microbial characteristics of IE vary by country and also by different centers in a single country, reflecting the local epidemiology of IE, the criteria of diagnosis adopted, the practice of starting antimicrobial drugs before blood collection, and the methods adopted for etiological diagnosis. The identification of pathogenic microorganisms is vital for choosing effective targeted antimicrobial treatment, which is a cornerstone of the treatment for IE.^[10-12]^

Data in literature of blood culture-negative on outcomes of IE patients are controversial. Investigations of the impacts of blood culture-negative on results of IE patients with nonsurgical and surgical intervention are rare.^[9,10,13,14]^ The objective of this research was to analyze impacts of blood culture-negative on results of IE patients with nonsurgical and surgical intervention.

## 2. Materials and methods

The experiment protocol for involving humans was in accordance to Helsinki Statement and national guidelines and was approved by the Medical Ethics Committee of The People’s Hospital of Guangxi Zhuang Autonomous Region. We completed a retrospective study of medical records of all IE patients between years 2006 and 2022. Diagnosis was based on modified Duke Criteria. The Ethical Committee of our institution approved the research.

### 2.1. Definitions

The blood culture-positive endocarditis was defined as IE with the identification of pathogenic microorganisms from blood cultures and/or tissue cultures from the excised valve or vegetation, whereas blood culture-negative endocarditis was defined as IE with negative blood cultures and negative tissue cultures.^[14]^

Time between symptoms and admission referred to duration between onset of symptoms and the date admitted to hospital.

Based on the classification of Global Results for Improvement in Kidney Disease (KDIGO), acute kidney injury is diagnosed if serum creatinine increases by ≥0.3 mg/dL (26.5 μmol/L) within 48 hours, serum creatinine is 50% higher than baseline in the first 7 days, or urine output is<0.5 mL/kg/h within 6 hours.^[15]^

Multiple organ failure (MOF) is considered a continuous process of different levels of organ collapse, instead of complete or absent events. The lungs, cardiovascular system, kidneys, liver, coagulating system, and central nervous system are considered the “key organs” that characterize MOFs.^[16]^

### 2.2. Primary outcome and the secondary outcomes

#### 2.2.1. Primary outcomes

The primary outcome was in-hospital mortality.

#### 2.2.2. Secondary outcomes

The secondary outcomes include all-cause mortality in follow-up and the incidence of complications, such as aortic regurgitation, mitral regurgitation (MR), tricuspid regurgitation, mean intubation time, ICU retention time, hospitalized time after surgery, postoperative chest drainage, fresh-frozen plasma, packed red cells, acute renal injury.

### 2.3. Variables

Parameters were investigated (Data in Supplementary Materials, Supplemental Digital Content).

### 2.4. Blood culture

Usually, multiple blood samples are collected when patients have fever to improve the accuracy of testing. Blood samples have to be taken from different sites at an interval of 6 to 12 hours if the patient is symptomatically stable and within 2 hours if there is hemodynamically labile. The strategy of blood culture interpretation and resorting to other criteria will be very crucial if the patient doesn’t clinically improve.

### 2.5. Two-dimensional Doppler echocardiography

Echocardiography was acquired by experienced cardiologists or technicians using standardized methods. LVEF was determined using the M-mode in the parasternal long-axis view or Simpson’s biplane method. Left ventricular end-diastolic diameter was collected using the American Society of Echocardiography guidelines. Left ventricular end-diastolic diameter was measured at end-diastole on parasternal views.

Two-dimensional transthoracic echocardiography is a first-line imaging of valve regurgitation, typically sufficient for diagnosis. Measure the reflux area using quantitative 2-dimensional echocardiography and proximal isokinetic surface area method. Collect color Doppler and continuous wave Doppler images of each blood flow condition using a Philips Sonos 7500 ultrasound system (Philips Medical Systems, Andover) equipped with an S3 multi frequency transducer. Due to the imaging window and transducer position being parallel to the blood flow, the ultrasound beam and reflux are well aligned.

### 2.6. Antibiotic treatment for infective endocarditis

The antibiotic treatment for infective endocarditis should follow the principles of early bactericidal agents, intravenous administration, and long course (4–6 weeks), and be adjusted according to drug sensitivity tests. The medication for autologous valve and artificial valve infections is different, and timely medical treatment and standardized medication are key. Core principles of antibiotic treatment include early use of fungicides, prioritize the use of antibiotics with strong bactericidal effects (such as penicillin, cephalosporin, vancomycin) to quickly control infections, intravenous administration as the main method, adequate drug concentration needing to be maintained through intravenous infusion, oral medication is only as an adjunct or for later maintenance, long course treatment, generally lasting 4 to 6 weeks, and adjustments based on drug sensitivity after identifying the pathogenic bacteria, selecting sensitive antibiotics specifically.

### 2.7. Follow-up

Echocardiogram, electrocardiogram, and X-ray chest film were investigated for all patients from discharge to date of death or the end date of the research once every 3 to 12 months. The patients were interviewed at the outpatient department or contacted by phone or WeChat at the last time follow-up.

### 2.8. Statistical analyses

We performed a normality test for all variates in the research by Kolmogorov–Smirnov test. We used the Wilcoxon rank-sum test for continuous variables. Continuous variables are presented as mean ± standard deviation if normally distributed and median (interquartile range) if not normally distributed. Continuous variables were analyzed using Wilcoxon rank-sum test if not normally distributed or Student *t* test if normally distributed. We investigated categorical data by Chi-square test or Fisher exact test. Logistic regression was used. We created Kaplan–Meier curves and compared the curves by log-rank test. All tests were 2-sided, with statistical significance defined by a *P* value of <.05. We completed the analyses by IBM SPSS version 24.0 software (IBM SPSS Inc., New York).

## 3. Results

### 3.1. Characteristics of the population with infectious endocarditis

2016 IE patients were enrolled into blood culture-negative group (n = 720) and blood culture-positive group (n = 1296). There were 720 blood culture-negative endocarditis patients (35.7%, 720/2016; Table 1).

**Table 1:** Characteristics of the patients.

Variable	Total (n = 2016)
Male, n	1402 (74.3%)
Age, yr	41.43 ± 17.12 (range, 18–82)
Body weight, kg	55.94 ± 12.22 (range, 13–90)
Time between symptoms and admission, mo	2.08 ± 1.93 (range, 0.1–12.3)
Vegetation size, mm	9.72 ± 6.77 (range, 0–29)
Aortic regurgitation, cm^2^	5.05 ± 7.12 (range, 0–33)
Mitral regurgitation, cm^2^	6.60 ± 6.37 (range, 0–27)
Tricuspid regurgitation, cm^2^	4.10 ± 4.81 (range, 0–20)
Left ventricular end-diastolic dimension, mm	58.06 ± 10.16(range, 29–84)
Left ventricular ejection fractions, %	62.37 ± 8.91 (range, 25–83)
Symptomatic neurological complications, n (%)	432 (21.43%)
Blood culture-negative, n (%)	720 (35.7%)

### 3.2. Comparison between blood culture-negative and -positive groups

Age (43.09 ± 14.64 versus 40.51 ± 18.30 years, *P* = .001), body weight (56.91 ± 9.07 versus 55.39 ± 13.63 kg, *P* = .008), time between symptoms and admission (2.48 ± 2.40 versus 1.86 ± 1.58 months, *P*<.001), vegetation size (10.97 ± 7.43 versus 9.03 ± 6.22 mm, *P*<.001), aortic regurgitation (5.83 ± 7.87 versus 4.61 ± 6.64 cm^2^, *P*<.001), aortic regurgitation ≥ 4 cm^2^ (48.6% versus 39.2%, *P*<.001), MR (6.99 ± 5.56 versus 6.39 ± 6.77 cm^2^, *P* = .045), MR ≥ 4 cm^2^ (68.5% versus 56.0%, *P*<.001), vegetation size ≥ 10 mm (53.3% versus 43.2%, *P*<.001), surgical patients (74.7% versus 21.3%, *P*<.001), time between symptoms and admission ≥ 2 months (51.1% versus 44.4%, *P* = .004), vegetations on aortic valve (46.7% versus 39.2%, *P* = .002), vegetations on aortic and mitral valves (15.6% versus 12.3%, *P* = .043), vegetations of congenital heart disease (6.7% versus 1.7%, *P*<.001), rate of surgical patients (74.7% versus 21.3%, *P*<.001), and in-hospital mortality (35.6% versus 17.3%, *P*<.001) in blood culture-negative group were significantly higher than those in blood culture-positive group (Table 2).

**Table 2:** Comparison between blood culture-negative and -positive groups.

Variable	Blood culture-negative group (n = 720)	Blood culture-positive group (n = 1296)	*P* value
Male, n	544 (75.6%)	928 (71.6%)	.056
Age, yr	43.09 ± 14.64	40.51 ± 18.30	.001
Body weight, kg	56.91 ± 9.07	55.39 ± 13.63	.008
Time between symptoms and admission, mo	2.48 ± 2.40	1.86 ± 1.58	<.001
Vegetation size, mm	10.97 ± 7.43	9.03 ± 6.22	<.001
Aortic regurgitation, cm^2^	5.83 ± 7.87	4.61 ± 6.64	<.001
Mitral regurgitation, cm^2^	6.99 ± 5.56	6.39 ± 6.77	.045
Tricuspid regurgitation, cm^2^	4.37 ± 4.50	3.95 ± 4.97	.058
Aortic regurgitation ≥ 4 cm^2^, n (%)	350 (48.6%)	508 (39.2%)	<.001
Mitral regurgitation ≥ 4 cm^2^, n (%)	493 (68.5%)	726 (56.0%)	<.001
Vegetation size ≥ 10 mm, n (%)	384 (53.3%)	560 (43.2%)	<.001
Symptomatic neurological complications, n (%)	160 (22.2%)	272 (21.0%)	.517
Time between symptoms and admission ≥ 2 mo, n (%)	368 (51.1%)	576 (44.4%)	.004
Left ventricular ejection fractions, %	62.61 ± 10.05	62.23 ± 8.21	.358
Vegetations on aortic valve, n	336 (46.7%)	512 (39.2%)	.002
Vegetations on mitral valve, n	235 (32.6%)	437 (33.7%)	.662
Vegetations on aortic and mitral valves, n	112 (15.6%)	160 (12.3%)	.043
Vegetations on tricuspid valve, n	32 (4.4%)	138 (10.6%)	<.001
Vegetations of congenital heart disease, n	48 (6.7%)	22 (1.7%)	<.001
Surgical patients, n	538 (74.7%)	276 (21.3%)	<.001
In-hospital mortality, n	256 (35.6%)	224 (17.3%)	<.001

### 3.3. Factors associated with blood culture-negative endocarditis

By univariate analysis, time between symptoms and admission ≥ 2 months (OR: 1.307, 95% CI: 1.089–1.569, *P* = .004), vegetation size ≥ 10 mm (OR: 1.502, 95% CI: 1.251–1.804, *P*<.001), aortic regurgitation ≥ 4 cm^2^ (OR: 1.467, 95% CI: 1.221–1.763, *P*<.001), MR ≥ 4 cm^2^ (OR: 1.705, 95% CI: 1.408–2.065, *P*<.001), vegetations on aortic valve (OR: 1.340, 95% CI: 1.115–1.610, *P* = .002), and vegetations of congenital heart disease (OR: 4.136, 95% CI: 2.476–6.910, *P*<.001) were found to be related to blood culture-negative endocarditis (Table 3).

**Table 3:** Factors associated with blood culture-negative status.

Model	OR	95% CI	*P* value
Univariate analysis			
Time between symptoms and admission ≥ 2 mo	1.307	1.089–1.569	.004
Vegetation size ≥ 10 mm	1.502	1.251–1.804	<.001
Aortic regurgitation ≥ 4 cm^2^	1.467	1.221–1.763	<.001
Mitral regurgitation ≥ 4 cm^2^	1.705	1.408–2.065	<.001
Vegetations on aortic valve	1.340	1.115–1.610	.002
Vegetations of congenital heart disease	4.136	2.476–6.910	<.001
Multivariate analysis			
Time between symptoms and admission ≥ 2 mo	1.339	1.112–1.612	.002
Vegetation size ≥ 10 mm	1.371	1.137–1.653	.001
Aortic regurgitation ≥ 4 cm^2^	1.333	1.103–1.612	.003
Mitral regurgitation ≥ 4 cm^2^	1.545	1.269–1.880	<.001
Vegetations on aortic valve	1.347	1.109–1.636	.003
Vegetations of congenital heart disease	4.478	2.655–7.555	<.001

CI = confidence interval, OR = odds ratio.

By multivariate analyses, time between symptoms and admission ≥ 2 months (OR: 1.339, 95% CI: 1.112–1.612, *P* = .002), vegetation size ≥ 10 mm (OR: 1.371, 95% CI: 1.137–1.653, *P* = .001), aortic regurgitation ≥ 4 cm^2^ (OR: 1.333, 95% CI: 1.103–1.612, *P* = .003), MR ≥ 4 cm^2^ (OR: 1.545, 95% CI: 1.269–1.880, *P*<.001), vegetations on aortic valve (OR: 1.347, 95% CI: 1.109–1.636, *P* = .003), and vegetations of congenital heart disease (OR: 4.478, 95% CI: 2.655–7.555, *P*<.001) were found to be related to blood culture-negative endocarditis (Table 3).

### 3.4. Analysis of the significance of blood culture-negative endocarditis

Univariate and multivariate analysis indicated that blood culture-negative is statistically associated with in-hospital mortality (*P*<.001), aortic regurgitation ≥ 4 cm^2^ (*P*<.001), MR ≥ 4 cm^2^ (*P*<.001), vegetation size ≥ 10 mm (*P*<.001), and mechanical ventilation length ≥ 72 hours (*P*<.001), respectively (Table 4).

**Table 4:** Analysis of the significance of blood culture-negative endocarditis.

Model		OR	95% CI	*P* value
Univariate analysis of risk factors of in-hospital mortality				
Blood culture-negative		2.640	2.141–3.257	<.001
Multivariable analysis of risk factors of in-hospital mortality				
Blood culture-negative		2.592	2.100–3.200	<.001
Univariate analysis of risk factors of aortic regurgitation ≥ 4 cm^2^				
Blood culture-negative	1.467		1.221–1.763	<.001
Multivariable analysis of risk factors of aortic regurgitation ≥ 4 cm^2^				
Blood culture-negative	1.313		1.081–1.594	<.001
Univariate analysis of risk factors of mitral regurgitation ≥ 4 cm^2^				
Blood culture-negative	1.705		1.408–2.065	<.001
Multivariable analysis of risk factors of mitral regurgitation ≥ 4 cm^2^				
Blood culture-negative	1.595		1.312–1.939	<.001
Univariate analysis of risk factors of vegetation size ≥ 10 mm				
Blood culture-negative	1.502		1.251–1.804	<.001
Multivariable analysis of risk factors of vegetation size ≥ 10 mm				
Blood culture-negative	1.372		1.133–1.661	<.001
Univariate analysis of risk factors of mechanical ventilation length ≥ 72 h				
Blood culture-negative	1.417		1.055–1.902	<.001
Multivariable analysis of risk factors of mechanical ventilation length ≥ 72 h				
Blood culture-negative	2.594		1.841–3.654	<.001

CI = confidence interval, OR = odds ratio.

### 3.5. Impacts of blood culture-negative status on the clinical outcomes of infectious endocarditis patients with surgical intervention

**Figure 1.**
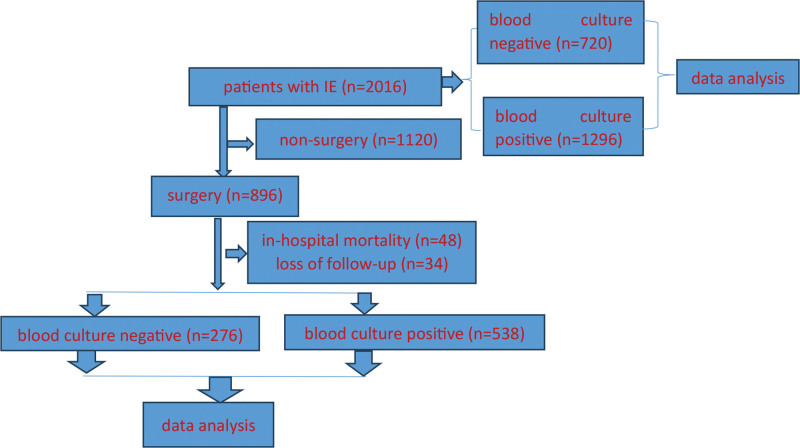
Flow chart.

Eight hundred ninety-six patients with IE who underwent cardiac operation during the research period were further divided into blood culture-negative (n = 272) and -positive (n = 624) subgroups. There were 48 in-hospital deaths (5.4%; Table 1, Fig. 1).

Vegetation size ≥ 10 mm (56.4% versus 35.3%, *P*<.001) and preoperative aortic regurgitation (6.30 ± 7.65 versus 5.17 ± 6.37 cm^2^, *P* = .022) in blood culture-negative subgroup were significantly higher than those in blood culture-positive subgroup (Table 5).

**Table 5:** Comparison of operative and follow-up results between blood culture-negative and -positive subgroups (n = 896).

Variable	Blood culture-negative subgroups (n = 272)	Blood culture-positive subgroups (n = 624)	*P* value
Preoperative			
Male, n (%)	176 (64.7%)	416 (66.7%)	.569
Age, yr	38.41 ± 15.70	38.53 ± 10.37	.909
Weight s, kg	55.86 ± 12.24	54.62 ± 10.92	.150
Time between symptoms and surgery, mo	2.13 ± 1.73	2.32 ± 1.06	.640
Vegetation size ≥ 10 mm	96 (35.3%)	352 (56.4%)	<.001
Preoperative LVEDD, mm	60.92 ± 9.61	61.41 ± 8.52	.469
Preoperative LVEF, %	62.03 ± 8.03	61.76 ± 7.00	.643
Preoperative aortic regurgitation, cm^2^	6.30 ± 7.65	5.17 ± 6.37	.022
Preoperative mitral regurgitation, cm^2^	7.01 ± 6.52	7.85 ± 5.42	.065
Preoperative tricuspid regurgitation, cm^2^	4.53 ± 5.00	4.98 ± 4.01	.197
Serum creatinine before surgery, μmol/L	82.10 ± 38.73	79.06 ± 16.75	.213
Operative			
Operative mortality, n	27 (9.9%)	21 (3.4%)	<.001
Aortic cross-clamp time	84.85 ± 35.55	83.56 ± 34.90	.065
Cardiopulmonary bypass time	156.67 ± 50.11	149.41 ± 54.65	.543
Mechanical ventilation length, h	52.47 ± 66.34	38.27 ± 41.55	.001
ICU retention time, d	4.77 ± 3.24	4.71 ± 2.22	.769
Hospitalized time postoperative, d	21.00 ± 9.53	18.05 ± 6.67	<.001
Serum creatinine 24 h after surgery, μmol/L	90.31 ± 45.11	84.12 ± 38.46	.049
Serum creatinine 48 h after surgery, μmol/L	109.64 ± 75.62	91.59 ± 48.97	<.001
Acute kidney injury postoperative, n	208 (33.3%)	64 (23.5%)	.003
Postoperative LVEDD, mm	48.08 ± 6.73	47.65 ± 7.71	.401
Postoperative LVEF, %	58.94 ± 7.08	58.88 ± 6.32	.905
Fresh-frozen plasma	612.82 ± 423.48	641.18 ± 555.21	.404
Packed red blood cells	2.81 ± 3.40	2.50 ± 2.66	.186

Follow-up	Blood culture-negative subgroups (n = 276)	Blood culture-positive subgroups (n = 538)	*P* value
Length of follow-up, mo	70.54 ± 54.35	66.85 ± 48.47	.341
All-cause mortality in follow-up, n	52 (18.8%)	81 (15.1%)	.167

LVEDD = left ventricular end diastolic diameter, LVEF = left ventricular ejection fractions.

Operative deaths (9.9% versus 3.4%, *P*<.001), mechanical ventilation length (52.47 ± 66.34 versus 38.27 ± 41.55 hours, *P* = .001), hospitalized time postoperative (21.00 ± 9.53 versus 18.05 ± 6.67 days, *P*<.001), serum creatinine 24 hours after surgery (90.31 ± 45.11 versus 84.12 ± 38.46 μmol/L, *P* = .049), serum creatinine 48 hours after surgery (109.64 ± 75.62 versus 91.59 ± 48.97 μmol/L, *P*<.001), and acute kidney injury postoperative (33.3% versus 23.5%, *P* = .003) in blood culture-negative subgroup were significantly higher than those in blood culture-positive subgroup (Table 5).

**Figure 2.**
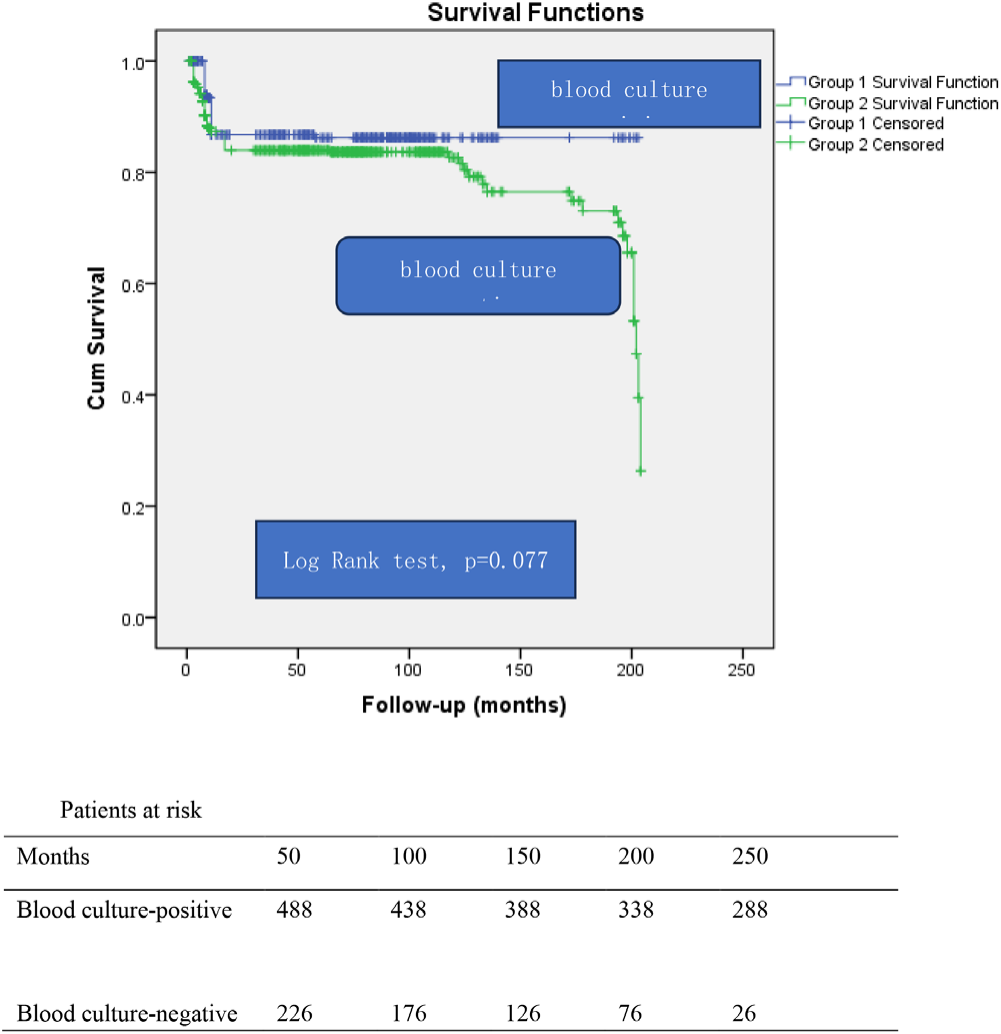
Kaplan–Meier curve. Blue line, group 1: group of blood culture-positive endocarditis; Green line, group 2: group of blood culture-negative endocarditis.

There is no statistically significant difference between blood culture-negative and -positive subgroups in terms of survival discharged from hospital (log-rank test, *P* = .077; Fig. 2).

## 4. Discussion

It is vital to early diagnose and identify IE pathogens and complete targeted antimicrobial treatment to better the prognosis of the disease and the health status of patients. Blood culture negative may be due to low bacteremia during blood draw, previous antimicrobial therapy, picky intracellular pathogens, or because the type of culture medium is not suitable for certain rare bacteria. According to reports, IE may be polymicrobial in up to 5% of cases. Identification of patient risk factors and detection of atypical pathogenic microorganisms may help develop more precise and effective treatment strategies based on clinical conditions.^[17-19]^

In-hospital mortality, aortic regurgitation ≥ 4 cm^2^, MR ≥ 4 cm^2^, vegetation size ≥ 10 mm, time between symptoms and admission ≥ 2 months, mechanical ventilation length, rate of surgical patients, and acute kidney injury postoperative in blood culture-negative group were significantly higher than those in blood culture-positive group. Time between symptoms and admission ≥ 2 months, vegetation size ≥ 10 mm, aortic regurgitation ≥ 4 cm^2^, MR ≥ 4 cm^2^ vegetations on aortic valve, and vegetations of congenital heart disease were found to be related to blood culture-negative. Univariate and multivariate analysis indicated that blood culture-negative is statistically significantly related to in-hospital mortality, aortic regurgitation ≥ 4 cm^2^, MR ≥ 4 cm^2^, vegetation size ≥ 10 mm, and mechanical ventilation length ≥ 72 hours, respectively. In our investigation, there were 720 blood culture-negative endocarditis patients (35.7%). In literature, the incidence rate of blood culture-negative endocarditis ranged from 2.5% to 70%.^[20-22]^

The pathogenic diagnosis of IE pathogens and fungi attaches to lesions formed before, forming vegetations getting along with platelets and fibrin, leading to leaflet perforation, leaflet prolapse caused by annulus destruction, or graft failure caused by vegetations, which can manifest as aortic and/or MR and cardiac failure. Vegetation may rupture with the influence of turbulence, pressure, and vegetation instability, forming embolism. These, in turn, may lead to sepsis, embolism, and ischemic areas in all kinds of organ systems.^[23-26]^ The causes of acute heart failure, cardiogenic shock, and MOF in patients with IE upon admission include aortic and MR caused by valve injury or papillary muscle and tendon rupture, myocardial dysfunction caused by ischemia due to coronary artery vegetation embolism or occlusion, myocarditis, cardiac conduction disorders, ventricular pseudoaneurysms, and sepsis. Heart failure, cardiogenic shock, and MOF caused by IE are independent predictors of mortality.^[27-30]^ Blood culture-negative was found to be significantly related to in-hospital mortality and clinical complications thus forming vicious circle. The vicious cycle must be broken by timely and effective targeted antimicrobial therapy.

The prognosis of patients with IE relies on timely and accurate diagnosis and treatment. It has been demonstrated that transesophageal tissue Doppler echocardiography expedites the detection of vegetations in IE. The implementation of transthoracic echocardiography with pulsed wave tissue Doppler imaging can improve the visualization and differentiation of intracardiac masses through different color coding of the pathological structure compared to surrounding tissue.^[31-33]^

Quick and accurate pathogen identification is important for diagnosing the etiology of IE, guiding antimicrobial selection and adjustment. Blood culture is now the standardized method for diagnosing IE, but sensitivity to IE of conventional blood culture is low mainly because of previous antimicrobial treatment and microbial sensitivity or inability to culture. It is reported that the incidence rate of blood culture-negative endocarditis ranges from 15% to 60%. The recent exposure to antimicrobial is the cause of most cases of blood culture-negative endocarditis. False negative rate high, long time of blood culture, delayed diagnosis and treatment have significant adverse effects on prognosis. Identifying infected organisms remains important for ensuring optimal antimicrobial treatment, and it is urgent to improve diagnostic accuracy and effectiveness through early intervention to reduce mortality rates in patients with IE.^[34-36]^

In our study, in-hospital deaths, mechanical ventilation length, and acute kidney injury postoperative in blood culture-negative group were evidently greater than those in blood culture-positive group, suggesting that diagnoses and/or evaluation for surgery in blood culture-negative endocarditis were more often completed later than in patients with blood culture-positive endocarditis, and after the incidence of complications of the heart or systems. It cannot be ruled out that the elevated incidence of cardiac failure and valve dysfunction is due to the unrecognized and lack of antimicrobial spectrum of pathogens in blood culture-negative endocarditis, possibly because of poor efficacy of antimicrobial therapy. Blood culture-negative endocarditis is a risk factor for in-hospital mortality related to advance and inappropriate antimicrobial treatment. Kong et al completed the greatest investigation with a large European registry, comparing the results of culture-positive and culture-negative endocarditis to get data of 1-year follow-up for 3113 cases. The 1-year mortality rate was evidently greater in the blood culture-negative endocarditis group and in the blood culture-negative endocarditis subgroup, surgery was significantly correlated with survival rate.^[37-39]^

Blood culture-negative endocarditis is a fatal disease with a high incidence rate and mortality. Rapid detection and identification of pathogenic pathogens are crucial for timely and targeted treatment. There are various reasons for blood culture-negative endocarditis, including: treatment with antimicrobial s before collecting blood cultures; Collection of suboptimal specimens; and/or infections caused by picky (such as *Streptococcus mutans*), intracellular (such as Burkholderia, Bartonella), or non-cultivable or difficult to cultivate organisms (such as Mycobacterium, *Tropheryma whipplei*, and fungi); and noninfectious causes. The diagnostic field has developed with the incidence of non-culture methods, such as serological testing, targeted and shotgun metagenomic sequencing, capable of identifying pathogens that have historically been difficult to find through traditional blood cultures. Because of validation and widespread use of these technologies outside of reference laboratories, their roles are becoming more and more evident.^[40-42]^

### 4.1. Perspectives

IE is a microbial infection on the cardia endocardium, often affecting the native or prosthetic valves. In the past few decades, the epidemiology and etiology of this disease have undergone significant changes. With the growth of the elderly people, the incidence rate of degenerative valvular disease and the use of prosthetic valves have elevated, being the most vital risk factor for susceptibility. The alteration epidemiology has resulted in a change in the potential microbiology in IE, with Staphylococcus replacing Streptococcus as the main pathogenic pathogen. Other rare microorganisms, such as *Streptococcus agalactiae*, *Pseudomonas aeruginosa*, *Burkholderia baicalensis*, and Brucella, have also appeared or reappeared. IE caused by these pathogens, especially *Staphylococcus aureus*, is usually related to serious results with high incidence rate and mortality. Molecular diagnostic techniques are considered breakthrough strategies for diagnosing IE and have been introduced into Duke University as the primary diagnostic criteria for early and timely guidance of clinical antimicrobial treatment.^[34-39]^

### 4.2. Limitations

The limitations of this research comprise its retrospective design, which may lead to bias of selection due to the retrospective nature of the research and the roles of our hospital as a tertiary referral center. Long term recruitment of patients may have adverse effects on the accuracy of the results. Prospective randomized controlled trials are needed, and plans to reduce the incidence rate and mortality of IE in-hospital are needed.

## 5. Conclusions

In our investigation, blood culture-negative status is associated with higher in-hospital mortality and more clinical complications including multiorgan failure at admission, aortic regurgitation ≥ 4 cm^2^, MR ≥ 4 cm^2^, vegetation size ≥ 10 mm, and mechanical ventilation length ≥ 72 hours in IE.

## Author contributions

**Conceptualization:** Jing-bin Huang.

**Data curation:** Jing-bin Huang, Chang-chao Lu.

**Formal analysis:** Chang-chao Lu.

**Investigation:** Jing-bin Huang.

**Resources:** Chang-chao Lu, Zhao-ke Wen.

**Software:** Zhai Huang, Chang-chao Lu.

**Supervision:** Zhai Huang, Zhao-ke Wen.

**Validation:** Zhai Huang, Chang-chao Lu.

**Writing – original draft:** Jing-bin Huang.

**Writing – review & editing:** Zhao-ke Wen.


