# Supplementary material for: Impacts of blood culture-negative status on outcomes of infectious endocarditis patients with nonsurgical and surgical intervention
Source: Medicine (Baltimore). 2026 May 8;105(19):e48760. doi: 10.1097/MD.0000000000048760 (PMC13166802; doi:10.1097/MD.0000000000048760)
Supplement: Supplementary file 1 [file medi-105-e48760-s001.docx]

Supplementary material

Variables were evaluated including gender (female/male), age, weight, time between symptoms and surgery, NYHA class, rheumatic heart disease, valvular heart disease, coronary heart disease, left ventricular end diastolic dimension, left ventricular ejection fractions, aortic regurgitation, mitral regurgitation, tricuspid regurgitation, serum creatinine, mean intubation time, ICU retention time, hospitalized time after surgery, postoperative chest drainage, fresh-frozen plasma, packed red cells, fluid balance on operation day, the first day following operation and the second day following operation, acute renal injury, multiorgan failure, long-term intubation, hepatic failure, respiratory failure, ventricular fibrillation, use of inotropic medication, blood lactate, extracorporeal membrane oxygenation (ECMO) requirement, and death.

**Abbreviations**

LVEDD =left ventricular end diastolic dimension；CT = computed tomography；CPB =cardiopulmonary bypass; ICU= intensive care unit; MOF=multiple organ failure ; ECMO= Extracorporeal Membrane Oxygenation; LVEF=left ventricular ejection fractions; AKI = acute renal injury; AKIN= acute kidney injury network；BMI (body mass index) = weight /(height^2^), (kg/m^2^); AVE = aortic valve endocarditis; ARA = aortic root abscess; PR = patch reconstruction; ARR = aortic root replacement; PVL = paravalvular leaks; PA = periannular abscess.
